# Full-Term, Unexpected, Abdominal Ectopic Pregnancy in a 38-Year-Old Patient With Leiomyomas

**DOI:** 10.7759/cureus.80066

**Published:** 2025-03-04

**Authors:** Rashid Sadek Azar, Jocelyn M Leon Turriza, Medardo Cohuo Muñoz, Nina Mendez Dominguez, Alondra Richaud Espinosa

**Affiliations:** 1 Obstetrics and Gynecology, Hospital General de Especialidades, Campeche, MEX; 2 Epidemiology and Biostatistics, Hospital Regional de Alta Especialidad de la Península de Yucatán, Campeche, MEX

**Keywords:** abdominal pregnancy, ectopic abdominal pregnancy, laparotomy decision, leiomyoma, pelvic mass, ultrasound imaging

## Abstract

Abdominal ectopic pregnancy is a rare and life-threatening condition, often leading to severe maternal complications. Its diagnosis is frequently challenging due to nonspecific symptoms and reliance on ultrasound findings that may not always indicate abnormalities. Case reports for this type of pregnancy are limited due to its low incidence.

We present a case of a 38-year-old patient from southern urban Mexico, undergoing her 3rd pregnancy. Around the due date, she was admitted to the obstetric emergency department, presenting with stabbing pain in the right lower extremity at the level of the thigh with perception of fetal movements and contracture of the right pelvic limb. After five hours without labor, an emergency cesarean section was performed. During intervention, the uterus (12 x 8 x 6 cm) was observed with the presence of a subserosa fibroid in the uterine fundus (1 cm), amniotic sac located in the abdominal cavity with normal amniotic fluid, and placenta with insertion in the posterior fundus, posterior aspect of the uterus and part of the sigmoid, with extension to the right broad ligament and distortion of the anatomy of the right appendix, which was removed with the help of surgery without complications.

We reported a rare case of ectopic pregnancy, which remained unexpected until cesarean section, due to a single ultrasound report indicating uneventful pregnancy and a healthy fetus. We conclude that an ectopic pregnancy can progress almost uneventfully and result in a neonate without anomalies.

## Introduction

Abdominal ectopic pregnancy is the second rarest type of ectopic pregnancy after cornual pregnancy. Implantation in the abdomen represents a challenge for the obstetrician-gynecologist since its low frequency and limited experience in its management complicate its treatment [1,2]. The incidence of abdominal ectopic pregnancy ranges from one in 1,000 to 30,000 pregnancies, representing 1% [2] of all ectopic pregnancies; it increases the risk of maternal mortality by seven to eight fold [1-5]. Newborns of abdominal pregnancies are at increased risk of limb and skull malformations, defects in the nervous system, and pulmonary hypoplasia [1-4]. Therefore, it is important to diagnose this rare type of pregnancy, with ultrasound being the gold standard, and to manage it effectively to reduce morbidity and mortality [4].

## Case presentation

A 38-year-old patient from an urban area in southern Mexico, currently in her third pregnancy, had two previous uneventful vaginal delivery pregnancies (10 and seven years prior) without the use of any contraceptive methods afterward. She denied having a history of ectopic or molar pregnancy, pelvic inflammatory disease, genital infections, or a history of assisted reproductive technology. Based on her last menstrual period, her gestational age was estimated at 40.2 weeks. She began prenatal care during the first trimester at a primary healthcare facility, attending seven follow-up visits. During one visit at our hospital at 23 weeks of gestation, she was discharged, and a subsequent ultrasound revealed an anterior fundal placenta, normal amniotic fluid levels, and a hyperechogenic oval-shaped lesion measuring approximately 28 mm on the left lateral uterine wall, suggestive of uterine leiomyomatosis.

Near her due date, she was admitted to the obstetric emergency department with stabbing pain in the right lower extremity at the thigh level, contracture of the right pelvic limb, and fetal movement perception. Her vital signs were stable. On examination, the uterine fundus measured 31 cm, and a single cephalic pregnancy was confirmed. The fetal heart rate was 138 bpm. Laboratory tests were within normal ranges, and the patient was deemed a candidate for labor induction. However, after five hours of being induced without labor progression due to irregular uterine activity, she was re-evaluated. A soft, centrally positioned cervix was noted, semi-effaced, with 2 cm of dilation and intact membranes. A mass was palpated between the cervix and vaginal canal, obstructing fetal passage.

**Figure 1:**
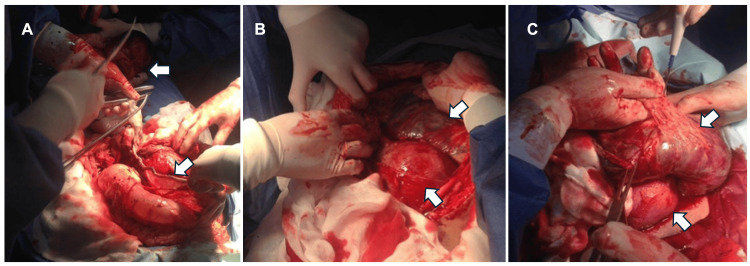
Trans cesarean section images. (A) Umbilical cord, full-term newborn, and part of the amniotic membranes attached to the intestine are observed. (B) A uterus of pre-pregnancy size is observed, and on its posterior aspect, the placenta is observed in the abdominal cavity. (C) Placenta extrauterine is observed with different adhesions.

An emergency cesarean section under balanced general anesthesia and a midline incision was performed. Intraoperatively, the uterus (12 × 8 × 6 cm) was found to have a 1-cm subserosal fibroid in the uterine fundus. The amniotic sac was located in the abdominal cavity (Figures 1A, 1B), with normal amniotic fluid. The placenta was implanted in the posterior fundus, extending to the posterior uterine aspect and partially involving the sigmoid colon (Figure 1C).

Additionally, the placenta extended into the right broad ligament, distorting the anatomy of the appendix, which was surgically removed using a gauze without complications. A thin, short umbilical cord was observed. Hemorrhage during surgery was the only complication. A live female newborn was delivered, weighing 3,300 grams, with Apgar scores of 3, 5, and 7 at one, five, and 10 minutes, respectively, where continuous positive airway pressure (CPAP) was applied at the beginning of treatment, and the baby was transferred to the NICU. The neonate had a gestational age of 40 weeks and a length of 49 cm. However, the neonate was discharged from the hospital after seven days in good health.

Postoperatively, the patient required intensive care management due to blood loss and the risk of cardiogenic shock. She was treated with adrenergic support and intravenous fluids. Her condition stabilized, and she was discharged seven days after surgery. At her six-week postpartum follow-up, no significant clinical or laboratory abnormalities were noted.

## Discussion

We report a rare case of ectopic pregnancy that remained undiagnosed until the cesarean section, despite a single ultrasound report indicating an uneventful pregnancy and a healthy fetus. Several maternal complications, such as hemorrhage and infection, can occur in such cases. In this patient, none of these complications were observed during gestation; however, hemorrhage was the only complication that occurred during the procedure.

The primary factor in determining maternal prognosis in cases of advanced abdominal pregnancy is the extent of placental tissue invasion and damage to surrounding structures. In our patient's case, both reproductive function and overall prognosis were favorable due to the successful removal of the placenta and minimal manipulation of extrauterine tissue during extraction. Postoperative follow-up with serial human chorionic gonadotropin (β-hCG) monitoring until remission was recommended for the patient.

A crucial takeaway from this case is the importance of imaging, regardless of ultrasound findings, in preventing similar occurrences. In resource-limited settings, patients and practitioners should be advised to provide ultrasound images whenever possible. Additionally, when images are unavailable, gynecologists must insist on obtaining in-hospital imaging studies to ensure accurate diagnosis and appropriate management.

## Conclusions

Based on the presented case, we conclude that an ectopic pregnancy can progress almost uneventfully and result in a neonate without anomalies. However, because the probability of a positive outcome is extremely low and the risks to both the mother and fetus are high, it remains crucial for first-contact physicians to provide thorough pregnancy surveillance. This includes regular assessments and sufficient ultrasounds to monitor for any anomalies. When a patient goes into labor without prior imaging to support a diagnosis, obtaining such images becomes of utmost importance. This case illustrates that a gestation in a uterus occupied even by a benign mass may not restrict fetal growth. However, the fetus may occupy adjacent structures. Therefore, patients with leiomyomatosis and related conditions should receive family planning counseling.
